# Radical Scavenging Capacity of Methanolic *Phillyrea latifolia* L. Extract: Anthocyanin and Phenolic Acids Composition of Fruits

**DOI:** 10.3390/molecules18021798

**Published:** 2013-01-30

**Authors:** Erol Ayranci, Naciye Erkan

**Affiliations:** Department of Chemistry, Faculty of Science, Akdeniz University, Antalya 07058, Turkey; E-Mails: eayranci@akdeniz.edu.tr

**Keywords:** antioxidant, anthocyanin, phenolic acid, phenylpropanoid, *Phillyrea latifolia* L., quercetin

## Abstract

Radical scavenging capacity of a crude methanolic extract from the fruits of *Phillyrea latifolia* L., commonly known as green olive tree or mock privet, was investigated with reference to anthocyanin standards, as flavonoids, and phenolic acid standards, as phenylpropanoids. Characterization with high performance liquid chromatography-diode array detection (HPLC-DAD) indicated the presence of keracyanin, kuromanin, cyanidin, ferulic acid, caffeic acid and rosmarinic acid at amounts of 289.1, 90.4, 191.4, 225.2, 221.2 and 190.1 mg/100 g fresh weight (FW) of fruits, respectively. Chlorogenic and *p*-coumaric acids were found to exist in lower amounts. Trolox equivalent antioxidant capacity (TEAC) and IC_50_ values of the plant extract were found to be 1.8 mM Trolox equivalents (TE)/g FW of fruits and 69.4 µg/mL, respectively, indicating the close radical scavenging activity of the extract to those of keracyanin and *p*-coumaric acid. The crude methanolic *P. latifolia* L. fruit extract was seen to be fairly potent in radical scavenging. Total phenolic content (TPC) of the plant extract was found to be 1652.9 mg gallic acid equivalent (GAE)/100 g FW of fruits.

## 1. Introduction

Free radicals are considered to be the species responsible for the development of many diseases including cardiovascular and neuro-degenerative diseases, and certain types of cancers. Regular consumption of formulations (herbal, vegetable or fruit extracts) or foods (fruits, vegetables, red wine, tea, whole grain cereals) rich in phenolic compounds, especially flavonoids, is reported to be related with reduced occurrence of these diseases [1,2]. Flavonoids can be subdivided into several classes: flavones, flavonols, flavanones, isoflavones, flavans, flavanols and anthocyanins. These compounds are widely distributed in plants. They contain a number of phenolic hydroxyl groups attached to ring structures, exerting antioxidant or radical scavenging activity through single electron or hydrogen atom donation [3].

Anthocyanins are members of a class of water-soluble plant pigments that can be classified chemically as both flavonoids and phenolics. Due to their particular chemical structure, they are characterized by electron deficiency, carrying a positive charge on the oxygen atom on the C ring. They are found in most land plants, fruits and berries, contributing colors to flowers and other plant parts. In addition to the capability of neutralizing free radicals, the resulting polyphenoxyl radical formed after scavenging other radicals must be stable through intramolecular hydrogen bonding upon further oxidation [4]. Compared to flavanols and flavanones, anthocyanin radicals are considered to provide this stability with their additional conjugation in the C ring. Anthocyanins [5,6] and extracts containing anthocyanins [7] were reported to show radical scavenging activity and lipid oxidation preventing ability. One of the major anthocyanin compounds existing in many of the fruit materials, cyanidin-3-*O*-glycoside, and its metabolites, were recovered from murine tissues which were proposed as targets for cancer chemopreventive intervention [8]. Anthocyanin concentrations achieved in the GI mucosa, prostate and the kidneys were found to be at a level high enough to show pharmacological activity in the same study.

Many plant species with significant antioxidant activities have been identified up to now. *Phillyrea latifolia* L., commonly known as green olive tree or mock privet, is an interesting one that has rarely been investigated. It is an evergreen shrub widely distributed in the Mediterranean region. Anthocyanin pigments of the fruits (berries) from the plant growing wild in Italy were reported to be found at considerably high levels [9]. Hydrogen peroxide scavenging activity and the effect on cytotoxic response of hepatocellular carcinoma cell lines of anthocyanins from *P. latifolia* L. fruits have been studied [10]. In Mediterranean Europe and North Africa, people have used infusions prepared from the leaves and fruits of *P. latifolia* L. as an astringent, diuretic and for the treatment of mouth ulcers and inflammations [11]. Flavonoid glycosides have been isolated from the leaves of the plant [12]. An extract of *P. latifolia* L. leaves was reported to show reductions in the elevation of the level of bilirubin and in the activity of alkaline phosphatase, both induced by CCl_4_ [13].

Most of the reports related to *Phillyrea latifolia* L. are associated with the leaves of the plant, as mentioned above. Radical scavenging capacity and flavonoid composition of fruits native to one geographical location have been rarely studied [9]. The purpose of the current study was to determine the *in vitro* radical scavenging capacity of a crude methanolic extract prepared from *P. latifolia* L. fruits, growing wild in Turkey, and to evaluate possible variations in antioxidant activity. Estimation of total phenolic content (TPC) and identification of anthocyanin and free phenolic acid components of the extract using HPLC-DAD with reference to authentic standards were also performed.

## 2. Results and Discussion

### 2.1. Anthocyanin and Free Phenolic Acid Analysis

**Figure 1:**
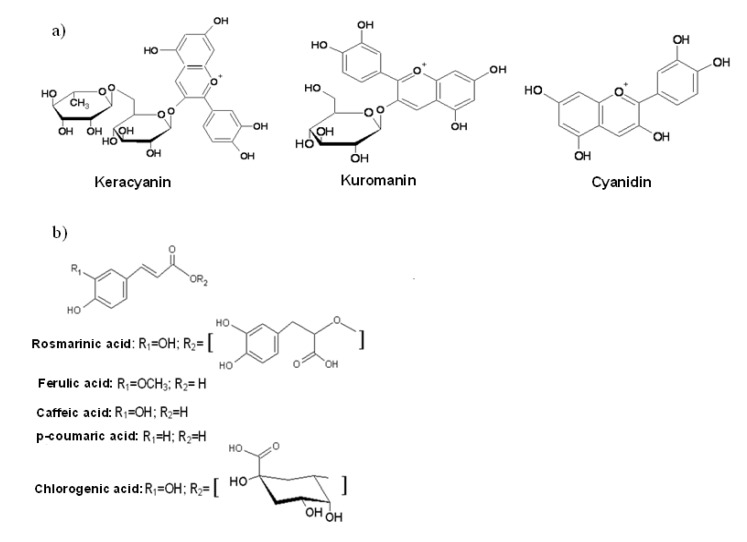
Chemical structures of (**a**) anthocyanins and (**b**) phenolic acids identified in *P. latifolia* L. fruits.

A crude methanolic extract from the fruits of *P. latifolia* L., growing wild in Turkey, was analyzed by HPLC-DAD for its anthocyanin components as flavonoids and phenolic acid components as phenylpropanoids. The purplish red color of the extract prompted us to assume that the fruits contain anthocyanin compounds beside other phenolics as this is the characteristic color of many components of this group. Anthocyanins in their glycosylated form with sugars were detected in the untreated methanolic extract whereas the other free phenolic compounds, especially the phenolic acids, were analyzed in the acid hydrolyzed extract. However, the phenolic compound standards in the bound form were unfortunately not available commercially. Quantification of peaks using standard compounds was done based on signals acquired at 520 nm for anthocyanins and 330 nm for free phenolic acids with an exception of *p*-coumaric acid, which was quantified at 280 nm. Conditions for chromatographic analysis of anthocyanins and phenolic acids were different from each other, as described in the experimental section. Anthocyanin components of interest, the structures of which are given in Figure 1a, were keracyanin (cyanidin-3-*O*-rutinoside), kuromanin (cyanidin-3-*O*-glycoside) and their hydrolysis product, cyanidin. Free phenolic compounds, other than anthocyanins detected in the methanolic plant extract, were all phenolic acids, namely chlorogenic, caffeic, *p*-coumaric, ferulic and rosmarinic acids, all of which are derivatives of hydroxycinnamic acid (Figure 1b). The extract was also analyzed for quercetin and apigenin, but these compounds could not be detected.

**Figure 2:**
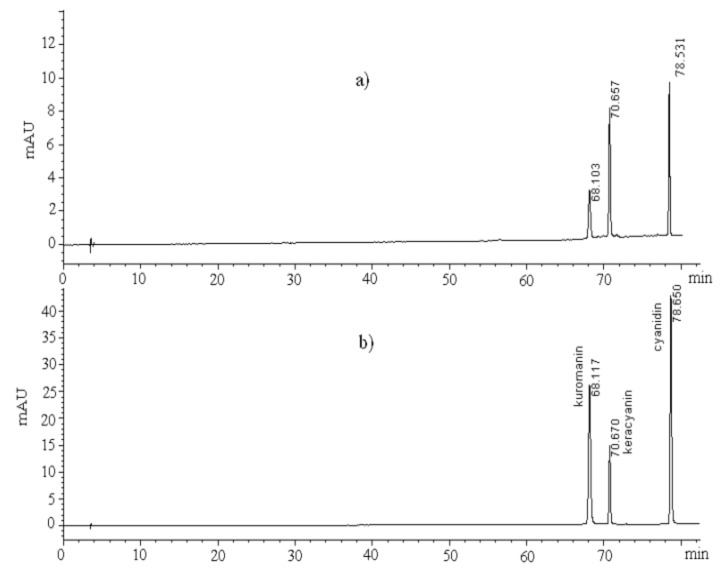
HPLC-DAD signals of (**a**) a crude methanolic extract from *P. latifolia* L. fruits for identification of its anthocyanin components, and (**b**) a standard anthocyanin mixture containing keracyanin, kuromanin and cyanidin obtained at 520 nm.

The chromatograms for the methanolic *P. latifolia* L. extract obtained from the fruits of the plant and for a standard anthocyanin mixture consisting of kuromanin, keracyanin and cyanidin, are given in Figure 2a,b, respectively. HPLC-DAD characterization based on retention time and UV-DAD detection with reference to authentic standards indicated the presence of these anthocyanin components in the extract.

**Figure 3:**
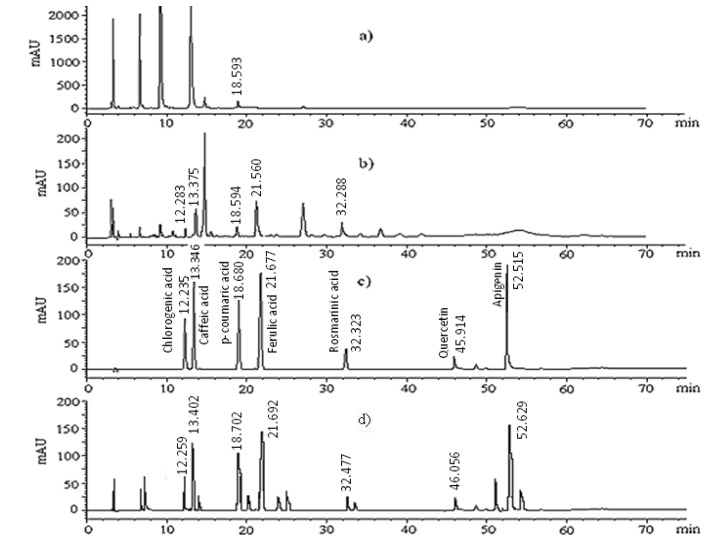
HPLC-DAD signals of (**a**) the acid-hydrolyzed methanolic extract from *P. latifolia* L. fruits obtained at 280 nm and (**b**) 330 nm, and (**c**) a standard mixture of phenolic compounds obtained at 330 nm and (**d**) the same standard mixture in (**c**) obtained at 330 nm after subjection to acid-hydrolysis.

Figure 3 represents HPLC-DAD signals of the acid-hydrolyzed methanolic extract obtained from *P. latifolia* L. fruits for characterization of phenolic acids at 280 nm and 330 nm. A hydrolysis step under mild conditions, which was found to be effective in preliminary studies, was applied to release aglycones from the glycosylated phenolic acids, in order to minimize their degradation as much as possible. Methanolic extract of *P. latifolia* L. fruits obtained in the present work was subjected to an acid-hydrolysis procedure, which had also been applied to a mixture of phenolic acid and flavonoid standards, containing chlorogenic, caffeic, *p*-coumaric, ferulic and rosmarinic acids, quercetin and apigenin in our previous study [14]. Recovery percentages of the individual components of this mixture were calculated, with respect to the peak areas obtained before (Figure 3c) and after (Figure 3d) the hydrolysis step and were included in Table 1. Recoveries were variable in the range from 63% to 87% (Table 1). These values can be considered as acceptable based on a previous report investigating different conditions of acid-hydrolysis for leafy vegetables containing phenolic acids, flavonols, flavones, glycosides and catechins [15]. Recovery values for anthocyanin components of the plant extract were not given in Table 1, as these components were analyzed in the untreated plant extract. Ferulic, caffeic and rosmarinic acids were detected in amounts of 225.2, 221.2 and 190.1 mg/100 g FW of fruits, respectively. Chlorogenic and *p*-coumaric acids were found to exist in lower amounts. Keracyanin, kuromanin and cyanidin, as anthocyanin components of the extract, were quantified in amounts of 289.1, 90.4 and 191.4 mg/100 g FW of fruits, respectively (Table 1).

Since the number of reports on the composition and radical scavenging activity of *P. latifolia* L. is limited, it is hard to compare our findings directly with other studies. A few reports to be mentioned in this regard are those by Longo *et al*. [9,10]. These researchers detected keracyanin and kuromanin in their extract, in amounts of 8.26 and 1.09 g/kg of *P. latifolia* L. fruits (berries), respectively. They isolated and used the same anthocyanin-rich extract obtained from *P. latifolia* L. fruits, in both studies. Keracyanin and kuromanin contents in our extract, can be given as ~2.89 and ~0.90 g/kg of fruits, respectively, in terms of the same unit reported by these researchers. The difference in amounts of individual anthocyanins detected, can be related to different extraction method applied for fruits in the previously mentioned studies and that applied in our study. Since our target was to obtain a whole phenolic fraction from the fruits, and not the anthocyanin fraction only, a general extraction method under reflux was applied, using methanol as a commonly used solvent in studies involving phenolic-rich fractions from natural sources [16]. No further purification has been applied for obtaining an anthocyanin-rich fraction. Therefore, such variations in the amounts of the components in the extracts are reasonable. The geographical location and the climate at which the fruits were grown, can also be counted as factors for the reported difference in the composition.

**Table 1:** Amounts and squared regression coefficients (R^2^) obtained from chromatographic analysis, correlation coefficients between DPPH**^•^** and ABTS**^•^**^+^ assay results and recovery percentages of individual phenolic compounds obtained from an acid-hydrolyzed standard mixture.

Analyte	Amount in the fruits ^a^ (mg/100 g FW)	Equation (y = mx); R^2^	Correlation coefficient ^b^between DPPH^•^ and ABTS^•+^assays	%Recovery ^c^
Kuromanin	90.4 ± 0.1E	y = 8.9628x;	0.9878 ± 0.0024	n.a.
		0.9902 ± 0.0007		
Keracyanin	289.1 ± 3.4A	y = 2.0798x;	0.9753 ± 0.0116	n.a.
		0.9922 ± 0.0017		
Cyanidin	191.4 ± 2.2C	y = 3.1801x;	0.9801 ± 0.0015	n.a.
		0.9933 ± 0.0014		
Chlorogenic acid ^d^	74.4 ± 1.7F	y = 17.515x;	0.9983 ± 0.0007	63.2
		0.9996 ± 0.0001		
Caffeic acid ^d^	221.2 ± 2.8B	y = 27.366x;	0.9996 ± 0.0047	70.6
		0.9999 ± 0.0001		
*p*-coumaric acid ^d^	112.7 ± 1.5D	y = 26.614x;	0.7684 ± 0.0125	71.3
		1.0000 ± 0.0000		
Ferulic acid ^d^	225.2 ± 2.6B	y = 27.178x;	0.9974 ± 0.0022	75.7
		0.9999 ± 0.0001		
Rosmarinic acid ^d^	190.1 ± 2.1C	y = 15.155x;	0.9608 ± 0.0248	67.8
		0.9996 ± 0.0000		
*P. latifolia* extract			0.9730 ± 0.0036	
Quercetin ^e^			0.8863 ± 0.0219	78.4

^a^, Means ± SD (standard deviation) (n = 3); means followed by the same letter within each column are not significantly different [LSD test, (*p* < 0.05)]. ^b^, Pearson correlation coefficient calculated based on % inhibition values provided by phenolic compound standards and the methanolic extract from *P. latifolia* L. fruits, as radical scavenging. ^c^, n.a.: not analyzed. ^d^, Amount in acid-hydrolysed *P. latifolia* L. extract. ^e^, Positive control.

### 2.2. Radical Scavenging Capacity

DPPH**^•^** and ABTS**^•^**^+^ scavenging assays are among the most frequently applied procedures for measuring antioxidant activity [17,18]. The TEAC and IC_50_ values of the methanolic extract of *P. latifolia* L. fruits and the individual phenolic compound standards contained in the extract are presented in Table 2. Some of the values for the standards (especially phenolic acids) were taken from our recent work [14], recalculated as mM Trolox equivalent (TE)/g of standard and included in this table to help interpretation of overall radical scavenging capacity of the extract since it was found to contain these phenolic acids beside anthocyanins. Quercetin was used as the positive control in the two radical scavenging assays. Total phenolic content of the fruits was found to be 1652.9 mg GAE/100 g FW of fruits. Methanolic *P. latifolia* L. extract obtained from the fruits of the plant provided TEAC and IC_50_ values of 1.8 mM TE/g FW of fruits and 69.4 µg/mL, respectively.

**Table 2:** Radical scavenging capacities of a methanolic extract from *Phillyrea latifolia* L. fruits and its anthocyanin and phenolic acid components, expressed as TEAC and IC_50_ values, based on ABTS**^•^**^+^ and DPPH**^•^** assays, respectively. Radical scavenging capacity ^a^.

Sample	TEAC ^b^ (mM TE/g FW of fruits or standard)	IC_50_ (µg/mL)
*P. latifolia* L. extract	1.8 ± 0.1 D	69.4 ± 5.8 B
Keracyanin chloride	1.7 ± 0.1 D	31.3 ± 3.4 D
Kuromanin chloride	3.9 ± 0.1 B	11.6 ± 2.1 E
Cyanidin chloride	4.1 ± 0.1 B	6.7 ± 0.7 F
Chlorogenic acid ^c^	0.9 ± 0.1 E	35.6 ± 2.1 D
Caffeic acid ^c^	3.9 ± 0.5 B	12.4 ± 0.7 E
*p*-coumaric acid^c^	1.9 ± 0.1 D	105.3 ± 4.3 A
Ferulic acid ^c^	3.0 ± 0.2 C	49.6 ± 2.3 C
Rosmarinic acid ^c^	3.2 ± 0.4 C	12.4 ± 0.8 E
Quercetin ^c,d^	4.8 ± 0.3 A	12.9 ± 1.8 E

^a^, Means ± SD (standard deviation) (n = 3); means followed by the same letter within each column are not significantly different [LSD test, (*p* < 0.05)]. ^b^, Data expressed as mM TE/g FW of fruits. ^c^, Results were taken from the study of Erkan *et al*. [14] and recalculated as mM TE/g of standard. ^d^, Positive control.

Many studies involving the antioxidant activities of phenolic compounds show that cyanidin glycoside members of flavonoids, as anthocyanins, have higher *in vitro* antioxidant activity than other members of phenolic compound classes [19]. Similar results were also obtained in our study. Cyanidin (TEAC: 4.1 mM TE/g of standard; IC_50_: 6.7 µg/mL) and kuromanin (TEAC: 3.9 mM TE/g of standard; IC_50_: 11.6 µg/mL) were observed to exhibit higher DPPH**^•^** and ABTS**^•^**^+^ radical scavenging capacities than almost all of the other phenolic compounds studied. Anthocyanins and their aglycones, anthocyanidins, especially cyanidin and delphinidin, were reported for their strong *in vitro* antioxidant activity in many studies [6,20]. Glycosylation of flavonoids is known to reduce their antioxidant activity [4]. Consistent with this, cyanidin, containing no sugar moiety, presented the highest activity among the anthocyanin compounds tested, although TEAC of kuromanin is statistically very close to that of cyanidin (*p* > 0.05). Since both keracyanin and kuromanin contain the same aglycone, the cyanidin molecule, the higher activity of kuromanin compared to keracyanin is probably due to the presence of a glucose molecule in kuromanin which is smaller than the rutinose group (a disaccharide) present in keracyanin. The approach of a free radical to a monosaccharide-containing molecule must be easier than to a larger disaccharide-containing molecule due to steric factors. Rosmarinic acid and caffeic acid were found to exhibit higher radical scavenging ability than other phenolic acid components of the extract (Table 2). Ferulic acid followed these two phenolic acids in this regard. Quercetin, as positive control, exhibited the highest TEAC value (4.8 mM TE/g of standard) among all phenolic compounds studied. It acted ~2.7 times stronger in scavenging ABTS**^•^**^+^ radicals than *P. latifolia* L. extract which is a complex fruit matrix and where interactions among various compounds may exist. It also presented a very low IC_50_ value (12.9 µg/mL), as well as kuromanin, caffeic acid and rosmarinic acid, indicating its very powerful antioxidative capacity. Since our crude extract may contain components other than phenolics that are soluble in methanol (e.g., sugars), radical scavenging capacity was lower than many of its individual components. However, the capacity is still very close to those of keracyanin and *p*-coumaric acid, and even higher than that of chlorogenic acid, as evaluated from TEAC values (*p* > 0.05), and significantly higher than that of *p*-coumaric acid, as evaluated from IC_50_ (*p* < 0.05) values (Table 2). The results of a recent study [21] showed that the anthocyanin fraction is mainly responsible for the total antioxidant capacity of red wines which is correlated with the electron transfer ability of these compounds. Longo *et al*. reported DPPH^•^ radical scavenging ability of the anthocyanin-rich fraction isolated from *P. latifolia* L. fruits to be around 50% when tested at a concentration of 0.005 mg/mL [10]. The same extent of radical scavenging was reached at a concentration of 69.4 µg/mL with our extract (IC_50_ value in Table 2). Some variations in the TEAC and IC_50_ values of the standard compounds can be originated from particular interactions between these compounds and the synthetic radical used in each assay media. Correlation values between DPPH^•^ and ABTS**^·^**^+^ assay results for individual phenolic compound standards and methanolic *P. latifolia* L. fruit extract, based on the percentage of the synthetic radicals scavenged (%inhibition), are also given in Table 1.

The presence of cyanidin in crude methanolic extract of *P. latifolia* L. fruits, at a considerably high level (Table 1), is probably due to the thermal breakdown of some of the glycosylated anthocyanins during the extraction procedure. Some cyanidin could also be released from its bound form in its natural environment and during harvest and storage time until being used in assays. Total phenolic content (TPC) of *P. latifolia* L. fruits (1652.9 mg GAE/100 g FW of fruits) can be considered to be high, compared to several fruits rich in anthocyanin fraction such as different blueberry cultivars (~264–528 mg GAE/100 g FW) [22] and bilberries (~759 mg/100 g FW) [23]. Unidentified peaks in the chromatographic analysis (Figure 3a and 3b) and the relatively high TPC value of the extract may indicate the presence of other possible phenolic compounds that need to be quantified.

## 3. Experimental

### 3.1. Materials

All solvents used were HPLC grade and purchased from Merck (Darmstadt, Germany). 1,1-diphenyl-2-picryl-hydrazyl (DPPH) radical, keracyanin chloride, kuromanin chloride, cyanidin chloride, caffeic acid, chlorogenic acid, *p*-coumaric acid, ferulic acid, rosmarinic acid, Folin-Ciocalteu reagent, Trolox and gallic acid were purchased from Sigma-Aldrich Chemie Gmbh (Munich, Germany). 2,2-azino-bis(3-ethylbenzothiazoline-6-sulfonic acid) diammonium salt (ABTS) was obtained from Fluka Chemie Gmbh (Stenheim, Germany). Deionized water (18 MΩ·cm) was used to prepare aqueous solutions. *Phillyrea latifolia* L., growing wild in nature, was collected from the campus area of Akdeniz University in December, 2011. The fruits were separated from the leaves and branches and left to dry in a convection oven at 27 °C ± 3 °C for a maximum of 3 h to remove any humidity at the outer surface of the fruits. The fruits were stored frozen at −20 °C until use.

### 3.2. Preparation, Acid Hydrolysis and Analysis of Methanolic Fruit Extract from *P. latifolia* L.

The whole fruits of the plant with their endocarp, mesocarp and the exocarp parts were blended in a blender and subjected to extraction under reflux for 3 h; 250 mL of methanol was used as the extracting solvent for 100 g of fruits. The mixture was filtered through Whatman paper (GF/A, 110 mm) and the solvent was removed by a rotary evaporator (Heidolph Laborota 4000 efficient, Schwabach, Germany) at 40 °C. The remaining extract was freeze-dried (−50 °C) by a freeze dryer (Operon FDB-5503, Seoul, Korea). The freeze-dried extract (~18.7 g) appeared in dark purplish-red color and was stored frozen at −20 °C until use.

An acid hydrolysis step was applied to the fruit extract that would be analyzed for the free phenolic acid components. The hydrolysis was performed according to the procedure reported previously [24]. Briefly, the plant extract (0.1 g) was incubated in a water bath at 80 °C for 50 min, after vortex-mixing with a mixture of 80% methanol (4 mL) and 4 M HCl (1 mL). The mixture was then purged with nitrogen so as to minimize the oxidation of phenolics. At the end of incubation, it was cooled to room temperature, made up to 5 mL with methanol and sonicated for 7 min. Then, the mixture was centrifuged at 3,000 × *g* for 10 min. The supernatant was separated and its solvent was removed completely by a rotary evaporator. A portion weighing 0.01 g from the remaining residue was diluted to 10 mL with methanol (approximately 1000 µg/mL). The solution was filtered through a 0.3 µm syringe filter (Millipore, Bedford, MA, USA) and subjected to analysis.

Untreated methanolic fruit extract of *P. latifolia* L. was used for the HPLC analysis of anthocyanin compounds using an Agilent 1100 series HPLC instrument (Agilent Technologies, Redwood, CA, USA) equipped with an auto sampler and a diode array detector (DAD). The column was a Hypersil ODS C_18_ type with 5 µm particle size; 4.6 × 250 mm i.d. used with Hypersil ODS 4.0 × 20 mm i.d. 5 µm guard cartridges. The mobile phase was composed of 5% ortho-phosphoric acid in H_2_O (solvent A) and 5% ortho-phosphoric acid in methanol (solvent B), and eluted at a flow rate of 0.9 mL/min. Gradient elution utilized were as: 0 min, 10% B, 40 min, 20% B, 60 min, 30% B and 80 min, 80% B. UV/VIS spectra were recorded every 2 s between 250 and 600 nm, with a bandwidth of 4 nm and the chromatograms were obtained at 520, 330 and 280 nm. Column temperature and injection volume were 28 °C and 7 µL, respectively. Anthocyanin standards, cyanidin, keracyanin (cyanidin-3-*O*-rutinoside) and kuromanin (cyanidin-3-*O*-glycoside), were diluted in methanol before analysis.

Acid-hydrolyzed methanolic fruit extract of *P. latifolia* L. was used for the HPLC analysis of free flavonoid compounds using the same chromatographic equipment mentioned above. The mobile phase was composed of 5% acetic acid in H_2_O (solvent A) and methanol (solvent B). It was eluted at a flow rate of 0.9 mL/min. Gradient elution utilized was: 0 min, 5% B; 5 min, 15% B; 25 min, 30% B; 39 min, 42% B; 47 min, 55% B; 50 min, 70% B; 56 min, 75% B and 60 min, 100% B. The chromatograms were acquired at 370, 330 and 280 nm. All other chromatographic conditions were the same as above. Free phenolic compound standards, chlorogenic, caffeic, ferulic, *p*-coumaric and rosmarinic acids, quercetin and apigenin were diluted in methanol before analysis.

Peak identification in HPLC analysis was performed by comparison of retention time and UV spectra of reference standards. Quantification of flavonoid components was achieved by injecting six diluted solutions (ranging between 5 and 100 µg/mL) for all standard compounds with three replicates for each of them at each concentration. Squared regression coefficients of calibration curves were calculated using Microsoft Excel within linear range of each compound. Quantification of individual components in the extract was done using the peak area of reference compounds and reported as mg/100 g FW of fruits.

### 3.3. Recovery of Acid-Hydrolysis Step

A model mixture was prepared from individual standards of chlorogenic acid, caffeic acid, *p*-coumaric acid, ferulic acid and rosmarinic acid, which were found to be the components of methanolic fruit extract of *P. latifolia* L., and quercetin and apigenin. This mixture was subjected to the same acid-hydrolysis procedure as the extract. Recovery of each component was calculated based on the amounts obtained from chromatographic analysis before and after the acid-hydrolysis step and expressed as percentage. The % recovery values were utilized in evaluating the sufficiency of the recovery upon acid hydrolysis of the extract.

### 3.4. DPPH• Radical Scavenging Assay

This assay was carried out as described by Blois [25] with some modifications. Various dilutions of the test materials (1.5 mL, 5–100 µg/mL for pure antioxidants; 25–250 µg/mL for the plant extract) was mixed with a 0.2 mM methanolic DPPH^•^ solution (1.5 mL). After an incubation period of 30 min at 25 °C, the absorbances at 515 nm were recorded as A_sample_ using a UV/VIS spectrophotometer (Cary 100 Bio). A blank experiment was also carried out applying the same procedure to a solution without the test material and the absorbance was recorded as A_blank_. The free radical scavenging activity of each solution was then calculated as percent inhibition according to the following equation:

% inhibition = 100 (A_blank_ – A_sample_)/A_blank_ (1)

Antioxidant activities of test compounds or the extract were expressed as IC_50_, defined as the concentration of the test material required to cause a 50% decrease in initial DPPH^•^ concentration. Quercetin was used as the positive control.

### 3.5. ABTS^•+^ Radical Scavenging Assay

This assay was performed based on the procedure described by Re *et al*. [26]. ABTS^•+^ radical cation was produced by reacting 7 mM aqueous ABTS with 2.45 mM (final concentration) potassium persulfate and keeping the mixture in the dark at room temperature for 16 h. Blue-green ABTS^•+^ was formed at the end of this period. The solution was diluted with ethanol to an absorbance of 0.70 ± 0.02 at 734 nm. Test materials were dissolved in and diluted with ethanol (50-300 µg/mL for the plant extract, 25–250 µg/mL for kuromanin, 10–250 µg/mL for keracyanin, 10–150 µg/mL for cyanidin, 10–100 µg/mL for chlorogenic acid, 5–25 µg/mL for caffeic acid, 10–100 µg/mL for *p*-coumaric acid, 5–100 µg/mL for ferulic acid, 5–50 µg/mL for rosmarinic acid and 5–50 µg/mL for quercetin) such that, after the introduction of an accurately measured volume of each dilution into the assay, they produced between 10%–90% decrease in the absorbance of the blank solution at 734 nm. After adding 100 µL of the test solution to 3.5 mL of ABTS^•+^ solution having A_734_ = 0.70 ± 0.02, absorbance was recorded at 6 min. Results were expressed as Trolox equivalent antioxidant capacity (TEAC), which was given by mM TE/g FW of fruits or g of standard. TEAC values were calculated using a separate concentration response curve (y = 0.1459x + 16.716; R^2^: 0.9949) for standard Trolox solutions (0.05; 0.1; 0.5; 1.0; 1.5; 2.0 mM). Corresponding TEACs of the plant extract and the standards were determined from regression equation of concentration response curve of Trolox. Quercetin was used as the positive control.

### 3.6. Total Phenolic Content (TPC)

TPC of *P. latifolia* L. fruit extract was determined using Folin-Ciocalteu reagent (FCR) according to the procedure reported by Singleton *et al*. [27] with some modifications. The extract was dissolved in deionized water to provide a concentration of 500 µg/mL. An aliquot of extract or deionized water (control) (0.5 mL) was mixed with FCR (0.5 mL) by manual shaking for 10–15 s. After 3 min, saturated Na_2_CO_3_ solution (0.5 mL) was added and the solution was diluted to 5 mL with deionized water. The reaction mixture was kept in the dark for 2 h and the absorbance was measured at 760 nm. The results were expressed in gallic acid equivalent (GAE) as mg GAE/100 g FW of fruits, which was determined utilizing a separately prepared absorbance versus concentration curve for gallic acid (ranging between 0.01 and 1 mM).

### 3.7. Statistical Analysis

The study was carried out using the same methanolic *P. latifolia* L. fruit extract as one replicate and all experiments for this extract were made as three repetitive measurements. The values were reported as means of the measurements with standard deviation (SD) values in tables. The data were analyzed using ANOVA methods of the general linear model (GLM) procedure of SAS software (Windows Release Version 7). Least significant difference (LSD) test was used to determine significant differences between mean values at α = 0.05. Pearson correlation analysis was also performed to display the correlations between results obtained from DPPH^•^ and ABTS^•+^ assays based on percent inhibition as radical scavenging.

## 4. Conclusions

A crude methanolic extract of *P. latifolia* L. fruits exhibited TEAC and IC_50_ values of 1.8 mM TE/g FW of fruits and 69.4 µg/mL, respectively. HPLC-DAD characterization indicated the presence of keracyanin, kuromanin and cyaniding, as anthocyanin components, in amounts of 289.1, 90.4 and 191.4 mg/100 g FW of fruits, respectively. Ferulic, caffeic and rosmarinic acids, as phenolic acid components, were detected in amounts of 225.2, 221.2 and 190.1 mg/100 g FW of fruits, respectively. Chlorogenic and *p*-coumaric acids were found to exist in lower amounts. *P. latifolia* L. fruits were found to have a quite high TPC value (1652.9 mg GAE/100 g FW), compared to several fruits rich in anthocyanin fraction. Fruits from *P. latifolia* L. can be an alternative natural source for obtaining extracts rich in phenolic compounds, especially anthocyanins and phenolic acids, bearing considerable radical scavenging capacity.
